# Beyond IDH-Mutation: Emerging Molecular Diagnostic and Prognostic Features in Adult Diffuse Gliomas

**DOI:** 10.3390/cancers12071817

**Published:** 2020-07-06

**Authors:** Kanish Mirchia, Timothy E. Richardson

**Affiliations:** Department of Pathology, State University of New York, Upstate Medical University, Syracuse, NY 13210, USA; richatim@upstate.edu

**Keywords:** glioma, astrocytoma, oligodendroglioma, glioblastoma, GBM, IDH, copy number variation, CNV, *EGFR*, *TERT*, *CDKN2A*

## Abstract

Diffuse gliomas are among the most common adult central nervous system tumors with an annual incidence of more than 16,000 cases in the United States. Until very recently, the diagnosis of these tumors was based solely on morphologic features, however, with the publication of the WHO Classification of Tumours of the Central Nervous System, revised 4th edition in 2016, certain molecular features are now included in the official diagnostic and grading system. One of the most significant of these changes has been the division of adult astrocytomas into IDH-wildtype and IDH-mutant categories in addition to histologic grade as part of the main-line diagnosis, although a great deal of heterogeneity in the clinical outcome still remains to be explained within these categories. Since then, numerous groups have been working to identify additional biomarkers and prognostic factors in diffuse gliomas to help further stratify these tumors in hopes of producing a more complete grading system, as well as understanding the underlying biology that results in differing outcomes. The field of neuro-oncology is currently in the midst of a “molecular revolution” in which increasing emphasis is being placed on genetic and epigenetic features driving current diagnostic, prognostic, and predictive considerations. In this review, we focus on recent advances in adult diffuse glioma biomarkers and prognostic factors and summarize the state of the field.

## 1. Introduction

Diffuse infiltrating gliomas are the second most common primary intracranial neoplasm, second only to meningioma. These tumors comprise approximately 22% of all central nervous system (CNS) neoplasms with more than 16,000 cases reported each year in the United States and a combined annual incidence of 5.13/100,000 individuals. In addition, glioblastoma (GBM) is the most common malignant CNS tumor, comprising 14.6% of all CNS tumors and 48.3% of all malignant CNS tumors with an average of 11,833 cases reported each year [1]. While these neuroepithelial tumors form a heterogenous group with marked variety in clinical behavior, genetic and pathologic features, prognosis, and response to treatment, they all tend to infiltrate as single cells into the surrounding non-neoplastic brain tissue and thus are typically considered to be surgically incurable, even when deemed “low-grade” [2]. Historically, these tumors have been classified based solely on their morphology and the proposed CNS precursor cell lineage into broad astrocytoma, oligodendroglioma, and oligoastrocytoma categories with subsequent grades based on histologic findings [3].

The 2016 WHO classification marked a significant departure from this previous morphology-alone classification, with the inclusion of chromosome 1p and 19q co-deletion as the “molecular signature” of oligodendroglioma and mutations in either *IDH1* or *IDH2* as the primary prognostic factor and molecular diagnostic criterion for adult astrocytomas [4,5]. The addition of molecular diagnostic factors has also served to all but remove the diagnosis of “oligoastrocytoma” except in situations where molecular analysis is unavailable [4,6]. 

Previous studies have shown in a grade-for-grade comparison within the astrocytoma group, IDH-mutant tumors have significantly longer progression-free survival (PFS) and overall survival (OS) compared to their IDH-wildtype counterparts [7,8]. As the vast majority of IDH mutations in gliomas occur in the *IDH1* R132 residue [9,10], immunohistochemical stains specific for mutant IDH1 R132H protein were developed as a molecular surrogate to aid in rapid and lower-cost detection of this key mutation [11,12], with the added benefit of assisting in differentiating between neoplastic and non-neoplastic astrocytes [13,14]. 

Subsequent work also revealed that the oncometabolite produced as a result of the *IDH1/2* mutation, 2-hydroxyglutarate (2-HG), can be detected by magnetic resonance spectroscopy [15,16,17], further solidifying the importance of this particular mutation, and molecular diagnostic techniques in general, in the future of glioma diagnosis. Although incompletely understood, IDH mutation appears to promote gliomagenesis, mediated through 2-HG and works in conjunction with additional molecular alterations including *ATRX* and *TP53* mutation to create the alternative lengthening of telomere (ALT) phenotype, as well as potentially promoting glial cell infiltration through HIF-1α and upregulating VEGF [18,19,20,21]. 2-HG also appears to induce widespread DNA hypermethylation [22,23], although this may affect certain chromosomal regions more than others and may differ somewhat by tumor type [24]. Additional modalities, such as methylation profiling, have emerged more recently to aid in classification with analysis of epigenetic markers unique to various tumor entities [25,26].

While IDH mutations have been established as one of the most important diagnostic and prognostic factors for adult diffuse gliomas, there remains significant intra-group heterogeneity in biologic behavior and clinical outcome in adult gliomas with significant survival outliers in all subtypes. As a result, a great deal of research effort has been spent searching for additional genetic and epigenetic prognostic factors to aid in further subdividing these tumors into more prognostically complete categories, as well as identifying new molecular targets for future therapies and understanding the underlying biology of these tumors. In hopes of addressing and better classifying these heterogenous tumors, research groups across the globe have reported a series of new molecular alterations and multivariate models. Additionally, numerous tumor entities have recently been discovered and defined by newly established recurrent molecular alterations [27]. Recent proof-of-concept studies have shown that a wealth of molecular data can be produced rapidly enough to be available at the time of histologic diagnosis [28,29], indicating that the technology is available for molecular data to guide treatment. What remains is the codification of specific molecular features in each subgroup of adult diffuse glioma to further refine glioma subgroups and guide pathologic and clinical decision making in specific sets of circumstances. 

In this review, we discuss recent advances made in understanding molecular biomarkers and prognostic factors identified since the publication of the 2016 WHO Classification of Tumours of the Central Nervous System, Revised 4^th^ edition, including those currently being codified into clinical recommendations under the new Consortium to Inform Molecular and Practical Approaches to CNS Tumor Taxonomy (cIMPACT-NOW) system [30,31]. We divide the gliomas discussed herein into 5 main histologic/molecular categories: IDH-mutant lower-grade astrocytoma (WHO grades II and III), IDH-mutant 1p/19q-codeleted oligodendroglioma (WHO grades II and III), IDH-mutant GBM (WHO grade IV), IDH-wildtype lower-grade astrocytoma (WHO grades II and III), and IDH-wildtype GBM (WHO grade IV), as well as various additional assorted gliomas not elsewhere classified.

## 2. IDH-Mutant Lower-Grade Astrocytoma

Lower-grade gliomas described here (Table 1) include both diffuse astrocytomas (histologic grade II) and anaplastic astrocytomas (histologic grade III), currently defined by increased mitotic activity [3,4], although recent studies have shown that there is no reliable mitotic count threshold that definitively separates these grades [32,33,34]. The vast majority of adult lower-grade astrocytomas harbor IDH mutations, although these are only rarely seen in pediatric cases [35]. In adults, mutations in *IDH1* or *IDH2* confer a significant survival benefit in histologically lower-grade astrocytomas compared to their IDH-wildtype counterparts, and as such, is the most important prognostic factor in this group [4]. IDH-mutant tumors also have more frequently increased nuclear p53 immunohistochemical staining and loss of nuclear ATRX reactivity, corresponding to more frequent mutations in *TP53* and *ATRX,* respectively. With only rare exceptions noted, these mutations are thought to be mutually exclusive with the 1p/19q co-deletion [6,36,37], aiding in distinguishing diffuse astrocytomas from oligodendrogliomas [38,39]. The vast majority of tumors with these histologic and molecular features have relatively benign clinical behavior; median post-surgical overall survival intervals range from 9.3 to 10.9 years in some cohorts, with variation based on patient age, tumor size and location, the extent of resection, post-surgical treatment, and other factors [34], although examples of outliers with rapid progression to GBM and short survival intervals have been identified in many reports [34,40,41,42].

Recent multi-dimensional genomic analyses [36,43,44] have revealed multiple pathways influencing prognosis and tumor response to therapy. One of the most significant alterations are genes in the retinoblastoma (RB) pathway, including *CDK4* on chromosome 12q14 and *CDKN2A* on chromosome 9p21. *CDK4* amplification and homozygous *CDKN2A* deletion have both been correlated with significantly shorter PFS and OS in multiple studies, and this effect was enhanced by the loss of 14q [43,44,45,46,47,48,49,50]. These alterations are mutually exclusive, which can be explained by the inactivation of the tumor suppressor *RB1* gene, requiring either amplification of *CDK4* (a cyclin-dependent kinase that inhibits RB1 activity) or homozygous deletion of *CDKN2A* (which encodes p16, a cyclin-dependent kinase inhibitor) [51]. Our analysis of a subset of astrocytoma cases within The Cancer Genome Atlas (TCGA) glioma dataset showed similar results with mutual exclusivity between *CDK4* and *CDKN2A* alterations in all except one case. *IDH*-mutant astrocytomas with either *CDK4* amplifications or *CDKN2A* deletions had significantly shorter survival (median PFS of 32 months; median OS of 36 months) compared to IDH-mutant astrocytomas without these alterations (median PFS of 95 months, *p* = 0.02; median OS of >172 months, *p* = 0.02) [52]. The association of mutation or homozygous deletion of *RB1* with shorter survival is not as well defined. Alterations in the PI3K-AKT pathway, including *PIK3R1* mutation, *PIK3CA* mutation, and *PDGFRA* amplification, have also been correlated with poor PFS and OS in cohorts of IDH-mutant astrocytomas [45,50]. New clinical guidelines from the cIMPACT-NOW group have codified biallelic *CDKN2A* deletions as a factor predicting poor clinical outcome in this group but have suggested that there is not enough evidence to definitively include other alterations, including *RB1* deletion or mutation, *CDK4* and *PDGFRA* amplification, or *PIK3R1* and *PIK3CA* mutations as independent prognostic factors at this time [53].

In other studies, isolated partial or complete loss of chromosome 19q with intact 1p has been shown to be associated with a better clinical outcome in IDH-mutant lower-grade astrocytomas but not in their GBM counterparts [54]. Nuclear factor erythroid 2-related factor 2 (*NRF2*) and *DJ1* are nuclear transcription factors that regulate intracellular antioxidants and phase II detoxification enzymes, protecting cells from oxidative damage. Increased nuclear Nrf2 and DJ1, as well as increased cytoplasmic levels of DJ1, are associated with better prognosis. However, increased cytoplasmic levels of Nrf2 have been shown to correlate with poor prognosis by an incompletely understood mechanism in IDH-mutant lower-grade astrocytoma cohorts [55]. IDH-mutant grade II-III tumors also show hypermethylation and silencing of oncogenes Insulin-like growth factor binding protein 2 (*IGFBP2*) and X-linked inhibitor of apoptosis (*XIAP*)-associated factor 1 (*XAF1*) [56,57,58,59]. Downregulation or epigenetic silencing of these genes independently correlate with better clinical outcome. The amplification of *MYCN* has also been correlated with shorter survival in some studies [49], although this was not consistent across studies [53]. Others have reported a shortened time to malignant progression with increased nuclear immunohistochemical expression of cMYC protein [60] and worse prognosis with increased nestin immunohistochemistry [61]. Multiple other specific genetic and immunohistochemical factors have also been considered, including c-MET, *EMP3*, *GSX2*, *EMILIN3*, *PPIC*, and *CHI3L1* [62,63,64], and multiple algorithms and multi-gene/multi-CNV panels have been suggested with varying technical complexity, practicality, and prognostic power [44,49,65,66,67,68,69]. These multi-gene models suggest that in many cases, there are alterations in multiple pathways acting in concert to determine the biologic behavior of the tumor, however, for practical purposes, these panels will likely need to be pared down to key alterations that can be identified soon after the initial histologic diagnosis. 

Multiple studies have also indicated that IDH-mutant lower-grade astrocytomas have significantly lower overall copy number variation (CNV; also referred to as copy number alteration, CNA), expressed as the sum of all gains and losses spread in a seemingly random pattern without regions of definitive “molecular signature” across the entire genome, compared to their IDH-mutant GBM counterparts [52,67] or IDH-wildtype lower-grade astrocytomas and GBMs [52]. It was observed that in retrospective studies, whole-genome copy number analysis performed on histologic grade II tumors with poor clinical outcome (measured as rapid progression to GBM and short overall survival intervals) showed significantly increased overall CNV at the time of initial resection compared to histologically similar tumors with less aggressive clinical courses [41,42,52]. As a clinical modality, CNV is relatively difficult to use compared to mutations such as *IDH1* R132H, in that it is a continuous variable instead of binary, thus a threshold of 15% (approximately 470 megabases) for whole-genome CNV was established for clinical prognostic purposes [70], although other groups have suggested a threshold of approximately 350 megabases [49]. 

Higher overall CNV correlated with worse prognosis within the IDH-mutant astrocytomas, irrespective of other histologic or genetic alterations. Cases with *CDK4* amplification or *CDKN2A* deletion and poor outcomes tend to have elevated CNV [52], although a significant portion of cases with high CNV and poor outcome did not have evidence of *CDK4* amplification or *CDKN2A* deletion [41,42], suggesting that these two measures may not be inextricably linked to one another. Measuring overall CNV appears to be a consistent and reproducible method for stratifying IDH-mutant astrocytomas. In addition, cohorts with high CNV (and poor clinical outcomes) also had an increased fraction of cases with chromothripsis [52] and mutations in genes with functions relating to maintenance of overall genome stability [41,52], suggesting a possible mechanism underlying both the elevated CNV and the poor clinical outcomes identified in these patients [71]. In addition, IDH-mutant astrocytomas with low G-CIMP methylation patterns (reduced global DNA methylation) have been associated with shorter overall survival [43,72]. cIMPACT-NOW update 5 guidelines include CNV and genomic instability as a possible prognostic factor, however, more research is needed to definitively assign a CNV threshold for clinical use [53].

In short, numerous targeted and large-scale studies have now demonstrated that molecular features are superior to histologic criteria in terms of diagnosis and prognosis of lower-grade IDH-mutant astrocytomas [4,36,41,43,46,52,66,73,74], however, unified and integrated terminology for combining the known genetic and epigenetic factors remains to be devised.

## 3. Oligodendroglioma

Oligodendrogliomas (Table 2) are now defined as an infiltrating glioma with the presence of mutation in either *IDH1* or *IDH2* and co-deletion of chromosomes 1p and 19q, regardless of other histologic features [4,76]. These tumors also typically lack the *ATRX* or *TP53* mutation [6,36,37,39], and have a generally good prognosis with significantly longer median PFS (3.9 years) and OS (14.9 years) than their non-co-deleted astrocytoma counterparts [77]; although molecular studies have demonstrated that there is significant heterogeneity within this group as well [78]. Whole 1p and 19q chromosomal arm loss is mediated through a t(1;19) (q10;p10) translocation [79,80] and is associated with improved response to chemotherapy [81]. These tumors also have frequent mutations in *CIC* and *FUBP1* (which are thought to be mutually exclusive with *TP53* and *ATRX* mutations) and the promoter region of *TERT* (*TERT*p) [82,83,84,85,86]. Unlike astrocytomas subgroups, mutations in *TERT*p (most frequently C228T or C250T) do not have a significant prognostic utility, due to the ubiquity of these mutations in adult oligodendrogliomas [87,88], although this feature is generally not seen in pediatric oligodendrogliomas [89,90]. The clinical significance of *CIC* and *FUBP1* mutations within the oligodendroglioma category is currently unclear.

Oligodendrogliomas have a minimum whole-genome CNV level of approximately 5.1%, which is accounted for by the losses of 1p and 19q, as well as frequent loss of chromosomes 4, 14, 15, and 18 [91]. In our own studies [92], cohorts of oligodendrogliomas with worse outcome (recurrence/progression within 36 months and/or death from the disease process within 60 months) had significantly higher overall CNV, although this was a smaller difference than our astrocytoma cohorts [41,42,52], but unlike astrocytomas [70], no clinically useful CNV threshold could be established. We also found a significantly higher total somatic mutation burden in oligodendrogliomas with poor clinical outcomes compared to those with longer, more conventional courses, and found that a total mutation burden of 1 mutation/MB (~30 total exonic mutations) could reliably separate oligodendroglioma cases into distinct good and poor prognostic categories by Kaplan-Meier analysis. In addition, both CNV and total mutation burden correlated positively with grade in oligodendrogliomas [92]. Relatively frequent gains in 7p and 11p as well as losses in 14q and 15q were associated with worse outcome and tumor progression, as were alterations in the Notch pathway and PI3K-AKT pathway, *NOTCH1*, *PIK3CA*, *PIK3R1*, and *ARID1A* [36,45,93]. Like their astrocytic counterparts, homozygous loss of *CDKN2A* has also been associated with poor outcomes in patients with anaplastic oligodendroglioma [94]. A number of studies have also shown that the polysomy of chromosomes 1 and 19 (defined as >2 signals detected by FISH for both 1q and 19p in 1p/19q co-deleted tumors) was associated with higher histologic grade, earlier recurrence, and worse patient outcomes compared to oligodendrogliomas without polysomy, although the negative impact on overall survival was not seen in all studies [95,96,97,98]. 

Although some studies have recently called the role of histologic features into question, in IDH-mutant 1p/19q co-deleted oligodendrogliomas histologic features remain an important prognostic factor with increased mitotic figures, microvascular proliferation (MVP), and necrosis dividing grade II from grade III [33,92,99,100,101]. Studies have shown survival differences within anaplastic oligodendrogliomas with and without MVP and necrosis [102], as well as other factors, including SSTR2A expression [103] and multi-gene scoring panels to designate low- and high-risk patients [104].

## 4. IDH-Mutant Glioblastoma 

IDH mutations are found in a minority of all glioblastoma specimens, although this molecular signature is far more frequent in what was called “secondary glioblastoma” in previous grading schemes [105,106,107]. Within a histologic grade, IV astrocytoma cohorts, akin to lower-grade astrocytomas, a mutation in *IDH1* or *IDH2* carries the most significant prognostic value [4,7] (Table 3). IDH-mutant GBMs show a median survival of 24–31 months (depending on treatment modalities) in comparison to 9–15 months for their histologically similar wildtype counterparts [4,7,106,108], although there are reports of much longer survival in some of these cases [109,110,111,112,113,114,115]. These tumors also occur at a younger age than their IDH-wildtype GBM counterparts (5^th^ decade compared to 7^th^ decade, respectively) [34,52,105,106], and have increased rates of *TP53* and *ATRX* mutation as well as lower rates of *TERT*p mutation, *PTEN* mutation, and *EGFR* amplification [107].

Multiple studies have demonstrated that the histologic features separating IDH-mutant glioblastoma from IDH-mutant lower-grade astrocytoma (microvascular proliferation and/or necrosis) remain important prognostic factors, as IDH-mutant glioblastoma has significantly shorter median recurrence-free and overall survival intervals than IDH-mutant grade II or III astrocytomas [4], and IDH-mutant GBMs tend to have higher whole-genome CNV as well as other more specific CNVs and mutations associated with tumor progression and malignancy than their lower-grade counterparts [36,52,67,73]. These tumors have more frequent instances of chromothripsis [52,67], increased frequency of mutation in genes responsible for overall genomic stability [52], and increased frequency of *CDK4* and *CDKN2A/B* alterations than their IDH-mutant lower-grade glioma counterparts, which corresponds to their worse clinical outcomes in terms of PFS and OS, but unlike within the lower-grade cohorts, we found no statistically significant prognostic effect of harboring these alterations in GBM [52]. Other groups have shown that *MET* amplification, *PDGFRA* amplification, and *CDKN2A* deletion are associated with poor prognosis within some IDH-mutant GBM cohorts [47,72,75,94]. Additional less-studied proposed factors include nuclear transcription factors that regulate cellular defense against oxidative damage, such as *SNRX1*, *NRF2*, and *DJ2*, which may have prognostic implications in IDH-mutant GBM cohorts in addition to IDH-mutant lower-grade astrocytomas [55].

In previous work, we have shown high-level amplification of the 12p13 locus containing the *CCND2* gene, which may be a predictor of favorable prognosis [115]. *CCND2* gene amplification has been associated with gemistocytic histology and, in some cases, slower cell proliferation [116]. *CCND2* amplification is not always correlated to increased transcripts or protein expression [117], and the mechanistic significance of the amplification is not currently understood, although the effect of *CCND2* amplification may be mediated through pep5, a Cyclin D2-derived peptide, which has been shown in some cancers to inhibit cell growth or cause cell death [118,119]. The D-type family of cyclins are modulators of cell cycle progression from G1 to S phase and induce cyclin-dependent kinases, including *CDK4* and *CDK6*. *CDK4* and *MDM2* (possibly *MDM4* as well) are frequently co-amplified due to location at the breakpoint-enriched region of 12q14-15 [120]. This co-amplification correlates with worse clinical prognosis and may be due to dense local chromosomal instability as well as initiation and progression of chromothripsis [120,121]. 

Like IDH-mutant lower-grade gliomas, more complex multi-gene and multi-CNV prognostic schemes have also been devised for the IDH-mutant GBM category [65,68,69], and large scale studies have shown that there are multiple genetic and epigenetic phenotypes that can result in longer survival in GBM cohorts [113].

## 5. IDH-Wildtype Lower-Grade Astrocytoma 

Like their IDH-mutant counterparts, IDH-wildtype lower-grade astrocytomas (Table 4) include tumors with a WHO histologic grade II or III, however, they have significantly worse progression-free and overall survival intervals after initial diagnosis and surgery [4,7,43]. Randomly selected IDH-wildtype grade II-III astrocytoma have statistically identical prognoses compared to IDH-wildtype glioblastoma [36,43,52], and many tumors originally diagnosed as IDH-wildtype lower-grade astrocytomas can now be separated into other diagnostic categories [124,125]. These lower-grade IDH-wildtype astrocytomas frequently have specific molecular alterations characteristic of IDH-wildtype glioblastoma, including high frequency of *EGFR* amplification, combined gain of chromosome 7 with loss of chromosome 10 (7+/10−), *TERT*p mutation, and tumors with these three factors have similar outcomes to IDH-wildtype glioblastoma, while lower-grade tumors without these characteristic alterations have significantly better clinical outcomes [36,43,88,126,127,128,129]. New cIMPACT-NOW update 3 clinical guidelines now recommend the diagnosis of “*diffuse astrocytic glioma, IDH-wildtype with molecular features of glioblastoma*“, for IDH wildtype astrocytomas lacking the histologic features of glioblastoma but having one or more of these “molecular grade IV” features [130], and this has been confirmed in subsequent validation studies [131].

There are, however, heterogenous subsets within this IDH-wildtype astrocytoma group, which show longer median survival, with a handful of proposed genetic alterations responsible for the better prognosis, including amplification of *MYB* and *BRAF* V600E mutation, which have both been shown to correlate with longer OS in select grade II IDH-wildtype gliomas [132]. In addition, age at initial presentation and mitotic index may also have a significant prognostic impact in this select group [33].

An additional subset of cases that may be included here is anaplastic astrocytoma with piloid features (AAPF) or high-grade astrocytoma with piloid features. These IDH-wildtype tumors are frequently found in the cerebellum with pilocytic astrocytoma-like features with increased peripheral invasion, increased mitotic figures, microvascular proliferation, and necrosis, and have a somewhat better prognosis than IDH-wildtype glioblastoma. These cases have a distinct methylation profile, and characteristically have mutations in the MAPK pathway, including *NF1*, *BRAF*, and *FGFR1* mutations, as well as frequent *ATRX* alterations and *CDKN2A/B* homozygous deletions [133,134,135].

## 6. IDH-Wildtype Glioblastoma 

Comprising the majority of “primary” or “de novo” glioblastoma (those occurring without a documented precursor lesion), IDH-wildtype GBMs (Table 5) characteristically have the worst clinical outcome and prognosis of all diffuse infiltrating gliomas in adults [4]. These tumors occur in older individuals compared to IDH-wildtype lower-grade astrocytomas or IDH-mutant glioblastomas, have shorter clinical history, and shorter post-surgical survival intervals [4,7,52,105,106], although long-surviving patients are represented in the literature [137]. Compared to IDH-mutant GBM cohorts, IDH-wildtype GBM had significantly more frequent instances of *EGFR* amplification, *PTEN* mutation, and *TERT*p mutation with significantly less frequent *ATRX* and *TP53* mutations [4,6,43,44,52,90,107]. While *TERT*p mutation occurs with similar frequency in IDH-wildtype glioblastoma and lower-grade astrocytoma, *EGFR* amplification and 7+/10− appear to occur more frequently in IDH-wildtype GBM [126,138].

While carrying an overall dismal prognosis, subsets of tumors with longer overall survival have been shown, with less frequent *EGFR* alterations and lower frequency of *CDK4* amplification or homozygous *CDKN2A/B* deletions. Other studies have shown that co-amplification of *CDK4* and *MDM2*, with or without chromosome 1, gain results in worse overall survival while isolated chromosome 1 or 19 gain without *CDK4*/*MDM2* co-amplification results in somewhat better survival in IDH-wildtype glioblastomas [139]. *PIK3CA* mutations [140] and combined *EGFR*, *PTEN*, and *CDKN2A* alterations [141] conferred worse prognosis in some IDH-wildtype glioblastoma subsets. In contrast to the effect of isolated *PTEN* protein loss in IDH-wildtype LGGs, within the IDH-wildtype GBMs, loss of this protein conferred a significantly better prognosis in one study [136]. The simultaneous gain of chromosomes 19 and 20 (19+/20+) has been shown to confer a survival benefit in patients with these tumors in univariate and multivariate analyses, although individual gains of either of these chromosomes (or partial chromosomes), did not have a similarly beneficial effect on prognosis [142].

Recently, we have shown that alterations in *EGFR*, 7+/10−, and *TERT*p mutations may have similar prognostic significance in IDH-wildtype GBM as in IDH-wildtype lower-grade tumors in both univariate and multivariate analysis [129] . There is significantly longer overall survival in tumors without any of these cIMPACT-NOW update 3 factors (GBM-C0) compared to tumors with at least one factor (GBM-C1-3). In addition, the GBM-C0 cohort appears to have statistically identical survival compared to IDH-wildtype lower-grade astrocytomas without cIMPACT-NOW 3 factors, and the GBM-C1-3 cohort has similar survival compared to the lower-grade tumors with at least one of these factors. In addition, there appears to be no significant prognostic difference amongst each of these three factors. Similar to previous studies [137,140], our results indicate that these long-surviving GBM patients are significantly younger and have lower overall somatic mutation burden. Additionally, 19+/20+ only conferred a prognostic benefit in the IDH-wildtype GBMs with at least one of the cIMPACT-NOW 3 factors [129] . 

Current guidelines recommend reflex testing for methylation of the promoter region of *O6-Methylguanine-DNA methyltransferase* (*MGMT*) in glioblastoma cases. *MGMT* promoter methylation status is an important predictive marker in terms of response to alkylating chemotherapeutic agents, specifically in IDH-wildtype GBM. The value of this in IDH-mutant GBM is less certain, although many studies demonstrate that *MGMT* promoter methylation is equally important in these tumors [122,123,143,144,145,146,147,148,149], and this beneficial effect of *MGMT* promoter methylation may in part depend on the presence of *TERT*p mutation [144,148,149]. MGMT is a DNA-repair protein whose expression levels inversely correlate with promoter methylation. Meta-analyses show patients with increased promoter methylation to have significantly longer overall but not progression-free survival. The MGMT protein also confers intrinsic resistance to temozolomide (TMZ) chemotherapy, along with decreased plasticity to aggressive adaptive tumor subtypes in response to TMZ therapy. *MGMT* promoter methylation has also been shown to correlate with methylation of the *XAF1* tumor suppressor gene promoter, leading to decreased XAF1 expression. XAF1 expression correlated inversely with patient survival (similar to its prognostic role in IDH-mutant LGG); thus, *XAF1* may have a unique, yet undescribed biologic function in astrocytomas [150]. Our own studies of IDH-wildtype GBM have demonstrated that while *MGMT* promoter methylation analysis is important in the vast majority of these cases, it may be of limited value in cases without at least one of the cIMPACT-NOW update 3 factors [127]. Other studies have noted alternative cellular strategies for TMZ resistance in GBM, including *MSH6* mutations occurring during TMZ therapy [151].

An additional area of investigation in GBM is immunotherapy, especially in light of the finding that the majority of current standardized GBM therapies (including radiotherapy and TMZ) tend to have an immunosuppressive effect. Similar to other systemic malignancies, programmed cell death-1 (PD-1) blockers and other immune checkpoint inhibitors have been utilized as monotherapy and in conjunction with other standard therapies to reverse local immunosuppression with mixed results. While most studies did not identify a benefit of anti-PD-1 therapy in randomized cohorts of GBM patients, others showed survival benefits in select groups, including some subsets of patients with hypermutated GBMs receiving neoadjuvant anti-PD-1 therapy [152,153,154,155]. As with many other tumor types, immunotherapy is an evolving field in GBM with some promising preclinical results and ongoing clinical trials. 

Of note, there are several histologically-defined variants of IDH-wildtype glioblastoma with characteristic genomic alterations and variation in outcome. Epithelioid glioblastoma is an IDH-wildtype variant with a relatively poor prognosis that harbors frequent *BRAF* V600E mutations, suggesting a possible connection to pleomorphic xanthoastrocytoma [156,157,158,159,160], although methylome heterogeneity exists within this group and it may represent multiple entities despite histologic similarity [161]. The *BRAF* V600E mutation (in addition to other *BRAF* alterations) is also found more frequently in other glioma subtypes, including pediatric diffuse gliomas and non-infiltrating tumors such as pilocytic astrocytoma and ganglioglioma. Recent studies have shown a response to BRAF inhibitors in some of these cases [162,163,164].

Giant cell GBM is another IDH-wildtype histologic variant with slightly better outcomes than other variants [165]. Cohorts of this GBM subtype have relatively infrequent *EGFR* amplification and *TERT* promoter mutation, but more frequent *TP53* and *ATRX* mutation [166]. Gliosarcoma is an IDH-wildtype, biphasic tumor with monoclonal glial- and mesenchymal-appearing components with poor prognosis, accounting for ~2% of all glioblastomas [167]. These tumors have similar genetic profiles to classic IDH-wildtype GBM with the exception of low rates of *EGFR* amplification [166].

IDH-wildtype tumors that show loss of ATRX immunohistochemical staining, frequently show mutations in histone H3 [168]. These mutations result in histone dysmethylation and dysregulation of gene expression on a global scale. H3K27M mutated tumors are more likely to occur in a central location (thalamus, brainstem, spinal cord) and in pediatric age groups. Due to their poor prognosis (median survival less than 6 months) and GBM-like behavior, they were recently classified as “Diffuse midline glioma, H3 K27M-mutant, WHO grade IV” [4,169]. Subsequently, tumors with this alteration have been identified in additional locations, including the third ventricle and pineal gland and in a broader age range than previously realized [170,171,172,173,174]. While less is known about this mutation in adult patients, there is evidence of worse outcome in adult gliomas with H3 K27M [175]. H3F3A/B (H3.3 G34R/V mutated protein) mutated tumors [176,177] comprise a histologically heterogeneous group of gliomas, usually in adolescent patients, with a marginally better clinical outcome compared to other IDH-wildtype GBM. These tumors show frequent *TP53* and *ATRX* mutation and characteristic lack of Olig2 expression. However, caution should be exercised in assigning grade IV based solely on the presence of an H3 K27M mutation since studies have shown its presence in lower grade tumors such as pilocytic astrocytoma and glioneuronal tumors or tumors in non-midline locations that do not necessarily have poor clinical outcomes [178,179,180,181].

## 7. Conclusions

This review highlights the speed of discovery of a vast variety of molecular alterations, as well as a better understanding of previously known genetic aberrations in diffuse gliomas. With wider adoption of high-throughput genetic sequencing, discoveries are being made at a faster pace than ever before, and a majority of the mutations we discuss here have been described in only the last 5–10 years. There are clearly survival outliers in all glioma classifications, some of which can be explained based on molecular factors reviewed here, while others certainly remain to be discovered. Codification of molecular markers and further stratification based on genetic and epigenetic features within these five main adult diffuse glioma groups discussed here and in other CNS tumors remains. While histologic examination is essential for initial tumor classification, expanded molecular testing will no doubt become the de facto standard of care.

For over a decade, efforts have been made to create therapies aimed at many of the molecular targets discussed in this review, including *EGFR*, members of the RB and PI3K-AKT pathways, and *IDH1/2* in the form of inhibitors, antibodies, vaccines, RNA-based therapies, and immunotherapy [21,182,183,184]. While many of these therapies found success in preclinical models, they have met with varying success in humans, although many are currently in clinical trials. In addition, many glial neoplasms have alterations in multiple pathways, making them resistant to single pathway targeted therapies. With future research efforts, identification and diagnostic incorporation of the next generation of molecular alterations will be necessary for better classification and prognostication, understanding the underlying biology, developing new targeted, biologically-relevant therapies and diagnostic modalities, as well as appropriate patient assignment to clinical trials in order to mitigate future costs and reap the most reward from trial data [66,139,149,185,186].

## Figures and Tables

**Table 1:** Prognostic factors in IDH-mutant histologically lower-grade astrocytoma.

Factor	Details	Reference
*IDH1/2* mutation	Definitional	[4,5,7]
1p/19q co-deletion	Incompatible with diagnosis	[4]
19q	Loss of 19q chromosomal arm correlates with better outcome	[54]
Total copy number variation (CNV)	Elevated CNV correlates with worse outcome	[41,49,52]
*CDK4*	Focal amplification correlates with worse outcome	[43,44,45,50,52]
*CDKN2A*	Homozygous deletion correlates with worse outcome, grade IV equivalent (cIMPACT-NOW update 5)	[48,49,50,52,53]
*PDGFRA, PIK3R1, PIK3CA*	Alteration correlates with worse outcome	[45,50,75]
*MYC*/*MYCN*	Focal amplification correlates with worse outcome	[49,60]
Nestin	IHC overexpression correlates with worse outcome	[61]

NOTE: Molecular alterations in white: Definitional; green: Overall positive effect on prognosis; red: Overall negative effect on prognosis.

**Table 2:** Prognostic factors in IDH-mutant, 1p/19q co-deleted oligodendroglioma.

Factor	Details	Reference
*IDH1/2* mutation	Definitional	[4,5,7]
1p/19q co-deletion	Definitional	[4,76,80]
*TP53*, *ATRX*	Mutation in either gene inconsistent with diagnosis	[4,6]
Total copy number variation (CNV)	Modestly elevated CNV correlates with worse outcome	[92]
Total mutation burden	Elevated mutation burden (>1 mut/MB; >30 total exomic mutations) correlates with worse outcome	[92]
1q & 19p	Simultaneous polysomy is correlated with worse outcome	[95,96,97,98]
*CDKN2A*	Homozygous deletion is correlated with worse outcome in anaplastic oligodendrogliomas	[94]
*ARID1*, *NOTCH1*, *PIK3CA*, and *PIK3R1*	Alterations in genes in Notch and PI3K pathways are associated with worse outcome	[36,45,93]

NOTE: Molecular alterations in white: Definitional; red: Overall negative effect on prognosis.

**Table 3:** Prognostic factors in IDH-mutant glioblastoma.

Factor	Details	Reference
*IDH1/2* mutation	Definitional	[4,5,7]
1p/19q co-deletion	Incompatible with diagnosis	[4]
*MGMT*	Promoter methylation predicts improved response to temozolomide	[122,123]
*CCND2*	Amplification is correlated with better clinical outcome	[115]
Total copy number variation (CNV)	Elevated CNV correlates with worse clinical outcome than IDH-mutant lower-grade gliomas, however, no in-group effects	[52,67]
*CDK4/MDM2*	Co-amplification is correlated with worse clinical outcome	[120,121]
*PDGFRA*	Amplification is correlated with worse clinical outcome	[75]

*CDKN2A*	Homozygous deletion is correlated with worse clinical outcome, especially when combined with MET amplification	[47,72,94]

NOTE: Molecular alterations in white: Definitional; green: Overall positive effect on prognosis; red: Overall negative effect on prognosis.

**Table 4:** Prognostic factors in IDH-wildtype histologically lower-grade astrocytoma.

Factor	Details	Reference
*IDH1/2* mutation	Incompatible with diagnosis	[4,5,7]
1p/19q co-deletion	Incompatible with diagnosis	[4]
Total copy number variation (CNV)	Elevated CNV correlates with worse clinical outcome than IDH-mutant lower- grade gliomas, however, no significant in-group effects	[52,127,129]
*EGFR* amplification, 7+/10−, *TERT*p mutation	Alteration of any of these factors correlates with worse clinical outcome; grade IV equivalent (cIMPACT-NOW update 3)	[126,129,130,131]
*PTEN*	Deletion correlates with worse clinical outcome	[136]
Nestin	IHC overexpression correlates with worse outcome	[61]

NOTE: Molecular alterations in white: Definitional; red: Over all negative effect on prognosis.

**Table 5:** Prognostic factors in IDH-wildtype glioblastoma.

Factor	Details	Reference
*IDH1/2* mutation	Incompatible with diagnosis	[4,5,7]
1p/19q co-deletion	Incompatible with diagnosis	[4]
*MGMT*	Promoter methylation predicts improved response to temozolomide, especially in the presence of TERT*p* mutation	[122,123,143,144,145,148]
Chromosomes 19 & 20	Co-gain correlates with better clinical outcome	[129,142]
Chromosomes 1 & 19	Isolated gain of either chromosome, in the absence of *CDK4/MDM2* co- amplification correlates with better clinical outcome	[139]
*BRAF* (V600E)	Mutation is correlated with better outcome	[152,153]
*EGFR* amplification, 7+/10−, *TERT*p mutation	Alteration of any of these factors correlates with worse clinical outcome; GBM-C0 status correlates with better clinical outcome	[129]
Total copy number variation (CNV)	Elevated CNV correlates with worse clinical outcome than IDH-mutant lower- grade gliomas, however, no in-group effects	[52,129]
*CDK4*/*MDM2*	Co-amplification is correlated with worse clinical outcome	[120,121,139]
*EGFR*/*PTEN*/*CDKN2A*	Co-alterations of these three genes is correlated with worse clinical outcome	[141]
*PIK3CA*	Mutations are correlated with worse clinical outcome	[140]
H3K27M	Mutation in midline glioma correlates with worse clinical outcome; grade IV equivalent (cIMPACT-NOW update 2)	[169,173,174,175,177]

NOTE: Molecular alterations in white: Definitional; green: Overall positive effect on prognosis; red: Overall negative effect on prognosis.
